# Emerging Research on the Relationship Between Diet, Gut Microbes, and Human Health

**DOI:** 10.3390/nu17162627

**Published:** 2025-08-14

**Authors:** Jennifer J. Barb, Gwenyth R. Wallen

**Affiliations:** Translational Biobehavioral and Health Promotion Branch, Clinical Center, National Institutes of Health, Bethesda, MD 20892, USA; grwallen@aol.com

The gut microbiome has emerged as a key player in nearly every aspect of human health, influencing not only physical well-being, but also emotional regulation and brain function, as well as dietary behaviors and cravings [1,2]. Increasing evidence implicates the reciprocal relationship between dietary behaviors and the gut microbiome as dietary patterns and specific nutrients can alter gut composition, function, and microbial diversity [3,4]. Microbial metabolites, such as short-chain fatty acids, bile acids, and neurotransmitter precursors, are now recognized as critical mediators of host–microbiome communication that influence immune function, metabolic regulation, and even cognitive and emotional processes [5,6]. Research exploring the complex interplay between diet and the gut microbiome is growing, revealing both promising insights and continued gaps across the literature. These studies are increasingly multidisciplinary, integrating microbiology, nutrition, immunology, and systems biology to advance precision health approaches [7,8]. This Special Issue, *The Relationship between Diet, Gut Microbes, and Human Health,* brings together six original research articles and one comprehensive narrative review across a broad array of microbiome research studies and aims to enhance our understanding of the relationship between diet, the microbiome, and human health. This editorial offers an overview of the key findings and emerging themes highlighted in this Special Issue.

## 1. An Overview of Published Articles and Themes

### 1.1. Theme 1: Microbiome-Informed Strategies to Address Malnutrition and Micronutrient-Related Disorders

Malnutrition, including both macronutrient and micronutrient deficiencies, remains a significant global health concern, affecting populations in low-income countries and individuals with chronic diseases in high-income settings [9]. In this context, Aristide Toussaint Nguélé et al. (contribution 1) investigated the relationship between helminth infection, malnutrition, and the gut microbiome in sub-Saharan Africa. Helminth infections, which impair nutrient absorption, are prevalent in low-resource settings and can exacerbate undernutrition. The study revealed location-specific differences between Pemba and Unguja Harbour, suggesting that ecological factors, in addition to diet, shape gut microbial profiles. Several genera, including *Akkermansia*, *Blautia*, *Dorea*, and *Odoribacter*, were negatively associated with infection and malnutrition, highlighting their potential as probiotic candidates. This study provided an interesting overview of the potential that targeting the gut microbiome may have as an integrative tool in improving health outcomes related to infection and undernutrition in high-risk populations.

Another study within this theme, conducted by Chai et al., examined the impact of zinc deficiency (ZD) on the gut microbiome in school-aged children (contribution 2). The researchers found that ZD was associated with significant alterations in gut microbial composition, including increased levels of some inflammatory and gut-disrupting metabolites. Notably, the probiotic *Bifidobacterium kashiwanohense* was reduced in children with ZD, and supplementation with this species showed potential to mitigate the microbiome imbalance in the study’s zinc-deficient population. These two studies implicated potential therapeutic interventions targeting malnutrition based on an endemic infection and micronutrient deficiencies by enhancing diets in specific foods rich in these micronutrients, as well as suggesting the potential importance of probiotics to target the gut microbiome to improve health outcomes.

### 1.2. Theme 2: Human Behavior and the Interplay of Diet on the Gut Microbiome

The gut–brain axis acts through bidirectional communication with both the central and enteric nervous systems involved including neural, hormonal, immune, and microbial signals. The impact of diet on the gut microbiome and how it may contribute to shaping human behavior has become a targeted area of microbiome research [10]. Jakobi et al. (contribution 3) examined adults with attention-deficit/hyperactivity disorder (ADHD) and reactive aggression, linking these traits to gut microbiota and dietary patterns categorized as either high energy, alcohol, or fiber. High energy intake was associated with higher aggression scores, and several bacterial genera were linked to both aggression and ADHD. The study suggested that the microbiome may mediate diet-related behavioral effects and reinforced the need for longitudinal studies, particularly in evaluating microbiota-targeted dietary interventions.

In a secondary exploratory analysis, Johnson et al. (contribution 4) evaluated the effects of time-restricted eating (TRE) on the gut microbiome in individuals with obesity. TRE is an eating pattern that limits food intake to a specific daily time window and this pattern has previously shown mixed effects on the gut microbiome in both preclinical and clinical studies [11,12]. The authors showed no significant differences in gut microbiome diversity or composition between the TRE group compared to controls. However, the authors noted that the small sample size, limited number of paired pre/post samples, and only having two time points may have reduced the study’s power. This intervention focusing on dietary behavior is of high interest given the mixed results from current studies. Future work should consider larger and varied samples as well as longer timeframes to assess microbial shifts and the impact of TRE.

### 1.3. Theme 3: Modern Diets and Microbial Disruption: Focus on Ultra-Processed Foods and Western Dietary Patterns

Ultra-processed foods (UPFs), which dominate the Western diet, have been increasingly associated with obesity, metabolic syndrome, and cardiovascular disease [13,14]. Few studies have directly examined how UPFs affect the gut microbiome, despite research examining their impact on health. To address this gap, Brichacek et al. (contribution 5) conducted a comprehensive narrative review of studies from the past decade that explored the relationship between UPF consumption and gut microbiota composition. In addition to summarizing current findings, the authors highlighted considerable variability in classification methods used to define UPFs across studies. Their review identified only four studies that directly assessed the impact of UPFs on the human gut microbiome. A major challenge noted was the inconsistency and lack of reproducibility in UPF classification systems, which limits the reliability of findings in this area. The authors emphasized the importance of refining classification approaches by accounting for factors such as food additive types and nutrient composition.

O’Sullivan et al. (contribution 6) conducted a microbiome study using an invitro colon called in vitro Mucosal ARtificial COLon (M-ARCOL) to examine a food borne pathogen often found in the Western Diet, Enterohemorrhagic *Escherichia coli* (EHEC), which causes diarrhea with the potential for life-threatening complications. The authors demonstrated the application of a complex in vitro model to test these life-threatening pathogens while providing insights into the diet, microbiota, and pathogen interactions in the human gut.

## 2. Closing

This Special Issue highlights diverse settings, populations, and research designs used to investigate the intersection of dietary behaviors and the gut microbiome. Three themes emerged from the contributed studies, and additionally, a translational gap in clinical nutrition practice was highlighted, specifically addressing a lack of knowledge among healthcare professionals by Mitsou et al. (contribution 7) regarding the gut–health interconnection and their understanding of prebiotics and probiotics. This gap reflects broader challenges in applying microbiome science to dietary guidance and clinical practice [15]. These articles collectively move the field forward, offering fresh insights while identifying gaps for future research. As interest in the microbiome continues to grow, a multidisciplinary and globally conscious approach will be key to realizing the full promise of microbiota-modulated dietary interventions for human health. As guest editors, we believe these contributions help to inform dietary research around the gut microbiome. We encourage future studies to adopt integrative, mechanistic, and diet lifestyle approaches for diet–microbe–health research.
